# The genome sequence of Ramsons hoverfly, *Portevinia maculata *(Fallén, 1817)

**DOI:** 10.12688/wellcomeopenres.20649.1

**Published:** 2024-02-19

**Authors:** Liam M. Crowley, Katie J Woodcock, University of Oxford and Wytham Woods Genome Acquisition Lab, Darwin Tree of Life Barcoding collective, Wellcome Sanger Institute Tree of Life Management, Samples and Laboratory team, Wellcome Sanger Institute Scientific Operations: Sequencing Operations, Wellcome Sanger Institute Tree of Life Core Informatics team, Tree of Life Core Informatics collective, Darwin Tree of Life Consortium

**Affiliations:** 1 Department of Biology, University of Oxford, Oxford, England, UK; 2 Tree of Life, Wellcome Sanger Institute, Hinxton, England, UK

**Keywords:** Portevinia maculata, Ramsons hoverfly, genome sequence, chromosomal, Diptera

## Abstract

We present a genome assembly from an individual *Portevinia maculata* (Ramsons hoverfly; Arthropoda; Insecta; Diptera; Syrphidae). The genome sequence is 1,125.3 megabases in span. Most of the assembly is scaffolded into 6 chromosomal pseudomolecules, including the X sex chromosome. The mitochondrial genome has also been assembled and is 18.98 kilobases in length. Gene annotation of this assembly on Ensembl identified 24,849 protein coding genes.

## Species taxonomy

Eukaryota; Metazoa; Eumetazoa; Bilateria; Protostomia; Ecdysozoa; Panarthropoda; Arthropoda; Mandibulata; Pancrustacea; Hexapoda; Insecta; Dicondylia; Pterygota; Neoptera; Endopterygota; Diptera; Brachycera; Muscomorpha; Eremoneura; Cyclorrhapha; Aschiza; Syrphoidea; Syrphidae; Eristalinae; Rhingiini; *Portevinia*; *Portevinia maculata* (Fallén, 1817) (NCBI:txid226160).

## Background

*Portevinia maculata* (Fallén, 1817), also known as the Ramsons hoverfly, is a Northern and Central European hoverfly species. It is widespread but localised in the UK ( Ball & Morris, 2015; van Veen, 2014). Individuals occur in woodlands and mature hedgerows, specifically associated with areas rich in Ramsons, *Allium ursinum* ( Ball & Morris, 2015). The larvae develop within the host plant bulbs and stem bases, hence their common name, the Ramsons hoverfly. It is a medium sized, dark hoverfly with silver-grey square shaped dust-spots on the 2 ^nd^ and 3 ^rd^ tergites ( Ball & Morris, 2015). The species additionally displays characteristic facial features including a rounded facial knob and bright orange antennae ( Stubbs & Falk, 2002). This set of distinctive morphological features along with their habitual presence in the proximity of Ramsons plants makes the species relatively straightforward to identify ( Ball & Morris, 2015).

Larvae are difficult to detect in bulbs until January to March time when they are actively growing and consequently have capacity to cause notable damage and discolouration to bulbs ( Rotheray, 1993; Stubbs & Falk, 2002). The adult flight period occurs from April to July, peaking in numbers from mid-May to early June, corresponding with when Ramsons are blooming ( Stubbs & Falk, 2002). The majority of records for the Ramsons hoverfly are of males found basking on sunny Ramsons leaves in woodland, frequently holding their wings in a distinguishing delta position ( Ball & Morris, 2015; Stubbs & Falk, 2002). Contrastingly, females are more elusive and are believed to spend a large proportion of time out of sight, close to the woodland floor amongst low growing foliage ( Ball & Morris, 2000; Ball & Morris, 2015; Stubbs & Falk, 2002). The chromosomally complete genome sequence for *Portevinia maculata* as part of the collaborative Darwin Tree of Life Project offers an opportunity to investigate and enhance our knowledge of this behaviourally and phenotypically distinct hoverfly species.

## Genome sequence report

**Figure 1.**
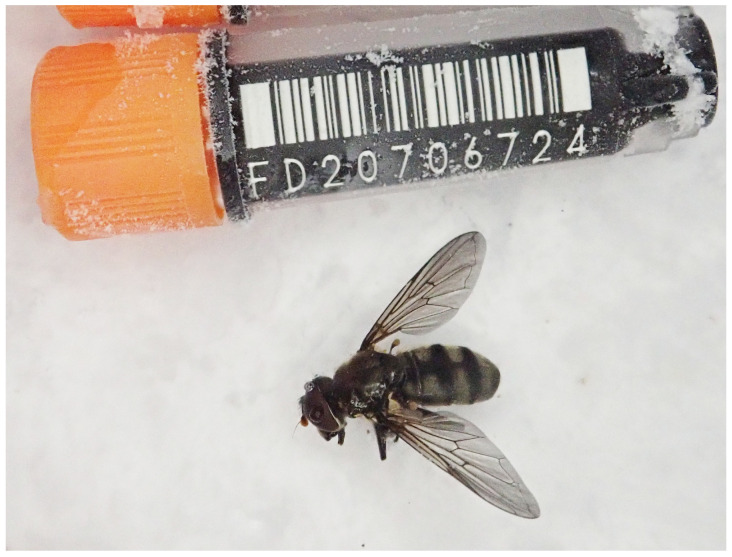
Photograph of the *Portevinia maculata* (idPorMacu1) specimen used for genome sequencing.

The genome was sequenced from one *Portevinia maculata* ( Figure 1) collected from United Kingdom | Berkshire | Wytham Woods (51.78, –1.34). A total of 29-fold coverage in Pacific Biosciences single-molecule HiFi long reads was generated. Primary assembly contigs were scaffolded with chromosome conformation Hi-C data. Manual assembly curation corrected 40 missing joins or mis-joins and removed one haplotypic duplication, reducing the scaffold number by 32.61%, and increasing the scaffold N50 by 76.35%.

**Figure 2.**
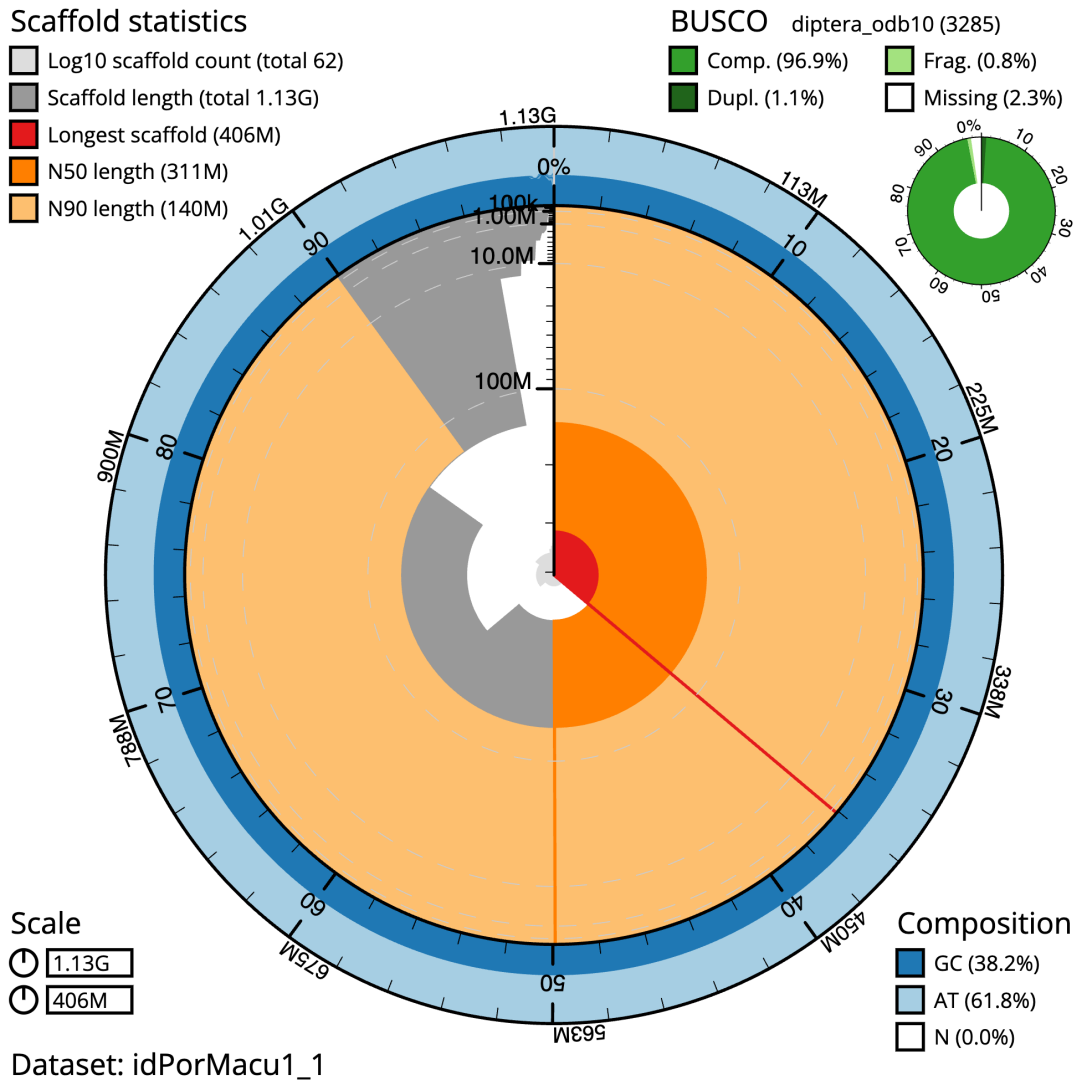
Genome assembly of *Portevinia maculata*, idPorMacu1.1: metrics. The BlobToolKit Snailplot shows N50 metrics and BUSCO gene completeness. The main plot is divided into 1,000 size-ordered bins around the circumference with each bin representing 0.1% of the 1,125,327,955 bp assembly. The distribution of scaffold lengths is shown in dark grey with the plot radius scaled to the longest scaffold present in the assembly (406,294,527 bp, shown in red). . Orange and pale-orange arcs show the N50 and N90 scaffold lengths (310,928,733 and 139,547,270 bp), respectively. The pale grey spiral shows the cumulative scaffold count on a log scale with white scale lines showing successive orders of magnitude. The blue and pale-blue area around the outside of the plot shows the distribution of GC, AT and N percentages in the same bins as the inner plot. A summary of complete, fragmented, duplicated and missing BUSCO genes in the diptera_odb10 set is shown in the top right. An interactive version of this figure is available at https://blobtoolkit.genomehubs.org/view/idPorMacu1_1/dataset/idPorMacu1_1/snail.

**Figure 3.**
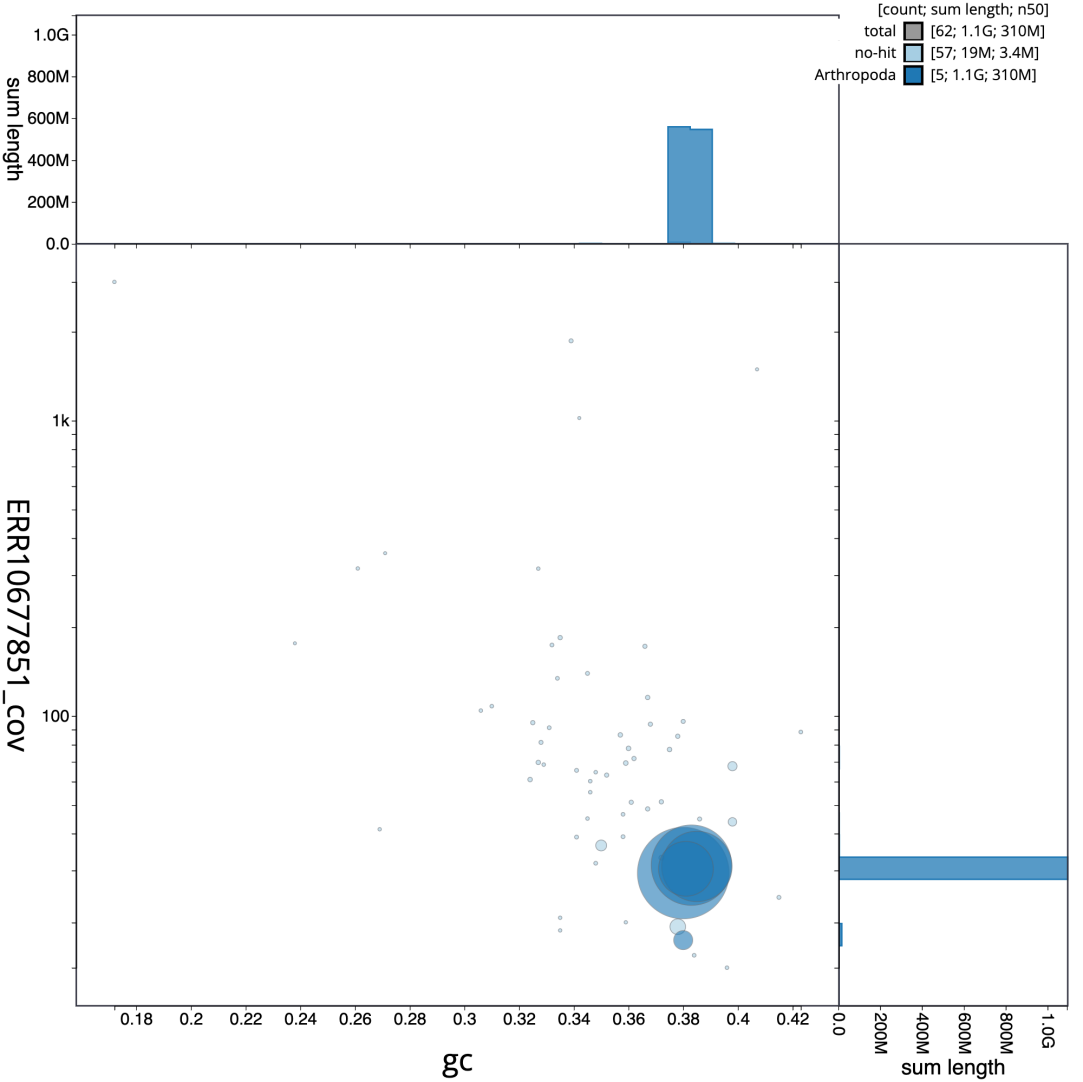
Genome assembly of *Portevinia maculata*, idPorMacu1.1: BlobToolKit GC-coverage plot. Scaffolds are coloured by phylum. Circles are sized in proportion to scaffold length. Histograms show the distribution of scaffold length sum along each axis. An interactive version of this figure is available at https://blobtoolkit.genomehubs.org/view/idPorMacu1_1/dataset/idPorMacu1_1/blob.

**Figure 4.**
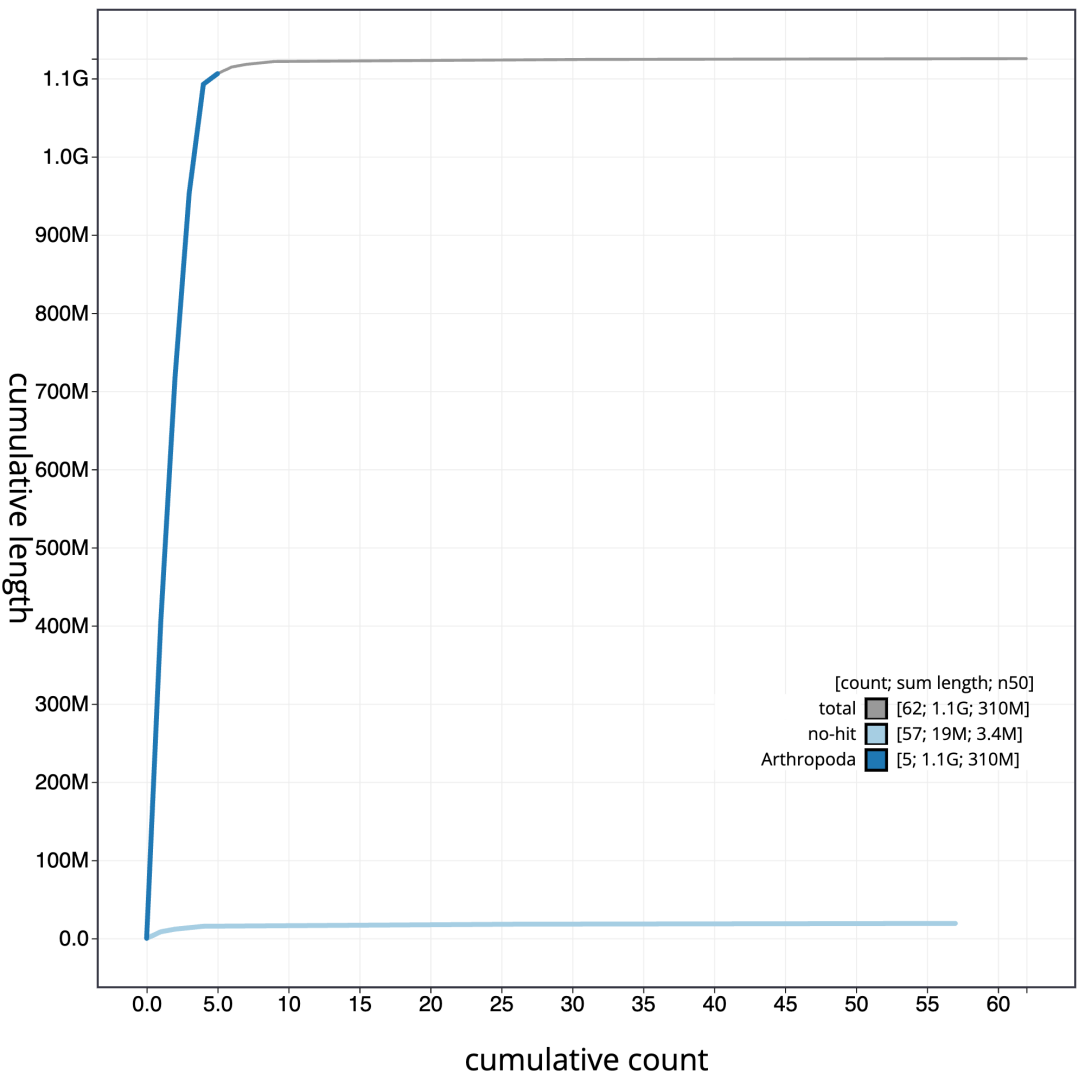
Genome assembly of *Portevinia maculata*, idPorMacu1.1: BlobToolKit cumulative sequence plot. The grey line shows cumulative length for all scaffolds. Coloured lines show cumulative lengths of scaffolds assigned to each phylum using the buscogenes taxrule. An interactive version of this figure is available at https://blobtoolkit.genomehubs.org/view/idPorMacu1_1/dataset/idPorMacu1_1/cumulative.

**Figure 5.**
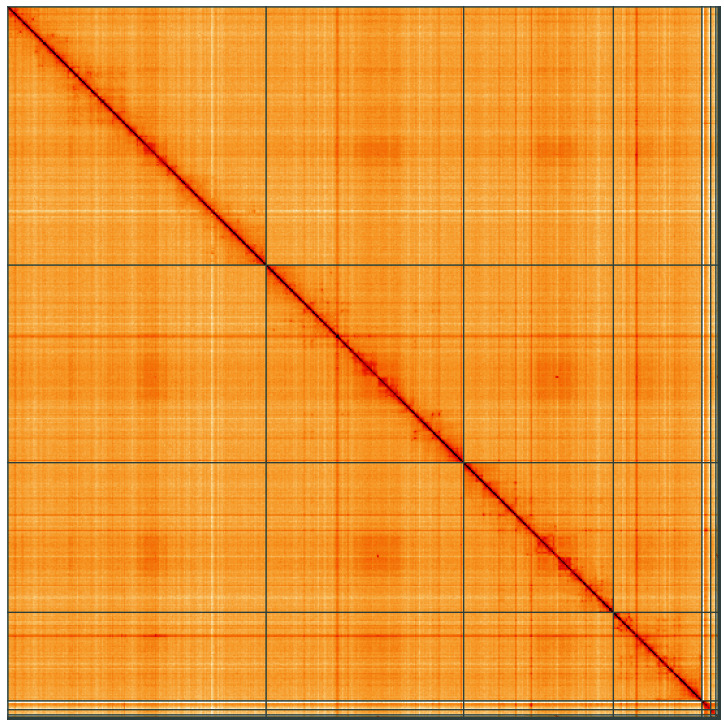
Genome assembly of *Portevinia maculata*, idPorMacu1.1: Hi-C contact map of the idPorMacu1.1 assembly, visualised using HiGlass. Chromosomes are shown in order of size from left to right and top to bottom. An interactive version of this figure may be viewed at https://genome-note-higlass.tol.sanger.ac.uk/l/?d=Otl7w1WoTVCakMcMc11ytQ.

The final assembly has a total length of 1125.3 Mb in 61 sequence scaffolds with a scaffold N50 of 310.9 Mb ( Table 1). The snailplot in Figure 2 provides a summary of the assembly statistics, while the distribution of assembly scaffolds on GC proportion and coverage is shown in Figure 3. The cumulative assembly plot in Figure 4 shows curves for subsets of scaffolds assigned to different phyla. Most (99.04%) of the assembly sequence was assigned to 6 chromosomal-level scaffolds, representing 5 autosomes and the X sex chromosome. Chromosome-scale scaffolds confirmed by the Hi-C data are named in order of size ( Figure 5; Table 2). The scaffolds making up Chromosome 5 have half coverage read mapping data. This may indicate that they are the Y chromosome. While not fully phased, the assembly deposited is of one haplotype. Contigs corresponding to the second haplotype have also been deposited. The mitochondrial genome was also assembled and can be found as a contig within the multifasta file of the genome submission.

**Table 1.** Genome data for *Portevinia maculata*, idPorMacu1.1.

Project accession data		
Assembly identifier	idPorMacu1.1	
Species	*Portevinia maculata*	
Specimen	idPorMacu1	
NCBI taxonomy ID	226160	
BioProject	PRJEB58242	
BioSample ID	SAMEA10166847	
Isolate information	idPorMacu1, thorax (DNA sequencing), head (Hi-C sequencing)	
Assembly metrics *		*Benchmark*
Consensus quality (QV)	65.2	*≥ 50*
*k*-mer completeness	100.0%	*≥ 95%*
BUSCO **	C:96.9%[S:95.9%,D:1.1%], F:0.8%,M:2.3%,n:3,285	*C ≥ 95%*
Percentage of assembly mapped to chromosomes	99.04%	*≥ 95%*
Sex chromosomes	X	*localised homologous pairs*
Organelles	Mitochondrial genome: 18.98 kb	*complete single alleles*
Raw data accessions		
PacificBiosciences SEQUEL II	ERR10677851	
Hi-C Illumina	ERR10684078	
Genome assembly		
Assembly accession	GCA_949715645.1	
*Accession of alternate haplotype*	GCA_949715845.1	
Span (Mb)	1125.3	
Number of contigs	323	
Contig N50 length (Mb)	7.9	
Number of scaffolds	61	
Scaffold N50 length (Mb)	310.9	
Longest scaffold (Mb)	406.29	
Genome annotation		
Number of protein-coding genes	24,849	
Number of gene transcripts	25,339	

* Assembly metric benchmarks are adapted from column VGP-2020 of “Table 1: Proposed standards and metrics for defining genome assembly quality” from Rhie *et al*. (2021). ** BUSCO scores based on the diptera_odb10 BUSCO set using version 5.3.2. C = complete [S = single copy, D = duplicated], F = fragmented, M = missing, n = number of orthologues in comparison. A full set of BUSCO scores is available at https://blobtoolkit.genomehubs.org/view/idPorMacu1_1/dataset/idPorMacu1_1/busco.

**Table 2.** Chromosomal pseudomolecules in the genome assembly of *Portevinia maculata*, idPorMacu1.

INSDC accession	Chromosome	Length (Mb)	GC%
OX454525.1	1	406.29	38.0
OX454526.1	2	310.93	38.5
OX454527.1	3	235.84	38.5
OX454528.1	4	139.55	38.0
OX454530.1	5	8.43	38.0
OX454529.1	X	13.57	38.0
OX454531.1	MT	0.02	17.0

The estimated Quality Value (QV) of the final assembly is 65.2 with *k*-mer completeness of 100.0%, and the assembly has a BUSCO v5.3.2 completeness of 96.9% (single = 95.9%, duplicated = 1.1%), using the diptera_odb10 reference set ( *n* = 3,285).

Metadata for specimens, barcode results, spectra estimates, sequencing runs, contaminants and pre-curation assembly statistics are given at https://links.tol.sanger.ac.uk/species/226160.

## Genome annotation report

The *Portevinia maculata* genome assembly (GCA_949715645.1) was annotated using the Ensembl rapid annotation pipeline ( Table 1; https://rapid.ensembl.org/Portevinia_maculata_GCA_949715645.1/Info/Index). The resulting annotation includes 25,339 transcribed mRNAs from 24,849 protein-coding genes.

## Methods

### Sample acquisition and nucleic acid extraction

A specimen of *Portevinia maculata* (specimen ID Ox001377, ToLID idPorMacu1) was netted in Wytham Woods, Oxfordshire, UK (latitude 51.78, longitude –1.34) on 2021-05-27. The specimen was collected and identified by Liam Crowley (University of Oxford) and preserved on dry ice.

The workflow for high molecular weight (HMW) DNA extraction at the Wellcome Sanger Institute (WSI) includes a sequence of core procedures: sample preparation; sample homogenisation, DNA extraction, fragmentation, and clean-up. In sample preparation, the idPorMacu1 sample was weighed and dissected on dry ice ( Jay *et al.*, 2023). Tissue from the thorax was homogenised using a PowerMasher II tissue disruptor ( Denton *et al.*, 2023a). HMW DNA was extracted using the Automated MagAttract v1 protocol ( Oatley *et al.*, 2023). HMW DNA was sheared into an average fragment size of 12–20 kb in a Megaruptor 3 system with speed setting 30 ( Todorovic *et al.*, 2023). Sheared DNA was purified by solid-phase reversible immobilisation ( Strickland *et al.*, 2023): in brief, the method employs a 1.8X ratio of AMPure PB beads to sample to eliminate shorter fragments and concentrate the DNA. The concentration of the sheared and purified DNA was assessed using a Nanodrop spectrophotometer and Qubit Fluorometer and Qubit dsDNA High Sensitivity Assay kit. Fragment size distribution was evaluated by running the sample on the FemtoPulse system.

Protocols developed by the Wellcome Sanger Institute (WSI) Tree of Life core laboratory are available on protocols.io ( Denton *et al.*, 2023b).

### Sequencing

Pacific Biosciences HiFi circular consensus DNA sequencing libraries were constructed according to the manufacturers’ instructions. DNA sequencing was performed by the Scientific Operations core at the WSI on a Pacific Biosciences SEQUEL II instrument. Hi-C data were also generated from head tissue of idPorMacu1 using the Arima2 kit and sequenced on the Illumina NovaSeq 6000 instrument.

### Genome assembly, curation and evaluation

Assembly was carried out with Hifiasm ( Cheng *et al.*, 2021) and haplotypic duplication was identified and removed with purge_dups ( Guan *et al.*, 2020). The assembly was then scaffolded with Hi-C data ( Rao *et al.*, 2014) using YaHS ( Zhou *et al.*, 2023). The assembly was checked for contamination and corrected as described previously ( Howe *et al.*, 2021). Manual curation was performed using HiGlass ( Kerpedjiev *et al.*, 2018) and Pretext ( Harry, 2022). The mitochondrial genome was assembled using MitoHiFi ( Uliano-Silva *et al.*, 2023), which runs MitoFinder ( Allio *et al.*, 2020) or MITOS ( Bernt *et al.*, 2013) and uses these annotations to select the final mitochondrial contig and to ensure the general quality of the sequence.

A Hi-C map for the final assembly was produced using bwa-mem2 ( Vasimuddin *et al.*, 2019) in the Cooler file format ( Abdennur & Mirny, 2020). To assess the assembly metrics, the *k*-mer completeness and QV consensus quality values were calculated in Merqury ( Rhie *et al.*, 2020). This work was done using Nextflow ( Di Tommaso *et al.*, 2017) DSL2 pipelines “sanger-tol/readmapping” ( Surana *et al.*, 2023a) and “sanger-tol/genomenote” ( Surana *et al.*, 2023b). The genome was analysed within the BlobToolKit environment ( Challis *et al.*, 2020) and BUSCO scores ( Manni *et al.*, 2021; Simão *et al.*, 2015) were calculated.

Table 3 contains a list of relevant software tool versions and sources.

**Table 3.** Software tools: versions and sources.

Software tool	Version	Source
BlobToolKit	4.2.1	https://github.com/blobtoolkit/blobtoolkit
BUSCO	5.3.2	https://gitlab.com/ezlab/busco
Hifiasm	0.16.1-r375	https://github.com/chhylp123/hifiasm
HiGlass	1.11.6	https://github.com/higlass/higlass
Merqury	MerquryFK	https://github.com/thegenemyers/MERQURY.FK
MitoHiFi	2	https://github.com/marcelauliano/MitoHiFi
PretextView	0.2	https://github.com/wtsi-hpag/PretextView
purge_dups	1.2.3	https://github.com/dfguan/purge_dups
sanger-tol/genomenote	v1.0	https://github.com/sanger-tol/genomenote
sanger-tol/readmapping	1.1.0	https://github.com/sanger-tol/readmapping/tree/1.1.0
YaHS	1.2a	https://github.com/c-zhou/yahs

### Genome annotation

The BRAKER2 pipeline ( Brůna *et al.*, 2021) was used in the default protein mode to generate annotation for the *Portevinia maculata* assembly (GCA_949715645.1) in Ensembl Rapid Release.

### Wellcome Sanger Institute – Legal and Governance

The materials that have contributed to this genome note have been supplied by a Darwin Tree of Life Partner. The submission of materials by a Darwin Tree of Life Partner is subject to the **‘Darwin Tree of Life Project Sampling Code of Practice’**, which can be found in full on the Darwin Tree of Life website here. By agreeing with and signing up to the Sampling Code of Practice, the Darwin Tree of Life Partner agrees they will meet the legal and ethical requirements and standards set out within this document in respect of all samples acquired for, and supplied to, the Darwin Tree of Life Project.

Further, the Wellcome Sanger Institute employs a process whereby due diligence is carried out proportionate to the nature of the materials themselves, and the circumstances under which they have been/are to be collected and provided for use. The purpose of this is to address and mitigate any potential legal and/or ethical implications of receipt and use of the materials as part of the research project, and to ensure that in doing so we align with best practice wherever possible. The overarching areas of consideration are:

•   Ethical review of provenance and sourcing of the material

•   Legality of collection, transfer and use (national and international)

Each transfer of samples is further undertaken according to a Research Collaboration Agreement or Material Transfer Agreement entered into by the Darwin Tree of Life Partner, Genome Research Limited (operating as the Wellcome Sanger Institute), and in some circumstances other Darwin Tree of Life collaborators.

## Data Availability

European Nucleotide Archive: *Portevinia maculata* (Ramson's hoverfly). Accession number PRJEB58242; https://identifiers.org/ena.embl/PRJEB58242 ( Wellcome Sanger Institute, 2023). The genome sequence is released openly for reuse. The *Portevinia maculata* genome sequencing initiative is part of the Darwin Tree of Life (DToL) project. All raw sequence data and the assembly have been deposited in INSDC databases. Raw data and assembly accession identifiers are reported in Table 1.

## Author information

Members of the University of Oxford and Wytham Woods Genome Acquisition Lab are listed here: https://doi.org/10.5281/zenodo.7125292.

Members of the Darwin Tree of Life Barcoding collective are listed here: https://doi.org/10.5281/zenodo.4893703.

Members of the Wellcome Sanger Institute Tree of Life Management, Samples and Laboratory team are listed here: https://doi.org/10.5281/zenodo.10066175.

Members of Wellcome Sanger Institute Scientific Operations: Sequencing Operations are listed here: https://doi.org/10.5281/zenodo.10043364.

Members of the Wellcome Sanger Institute Tree of Life Core Informatics team are listed here: https://doi.org/10.5281/zenodo.10066637.

Members of the Tree of Life Core Informatics collective are listed here: https://doi.org/10.5281/zenodo.5013541.

Members of the Darwin Tree of Life Consortium are listed here: https://doi.org/10.5281/zenodo.4783558.
