# Supplementary material for: The genome sequence of Ramsons hoverfly, Portevinia maculata (Fallén, 1817)
Source: Wellcome Open Res. 2024 Feb 19;9:52. [Version 1] doi: 10.12688/wellcomeopenres.20649.1 (PMC11391197; doi:10.12688/wellcomeopenres.20649.1)
Supplement: Peer review history

# Reviewer report: Reviewer response for version 1

**DOI:** 10.21956/wellcomeopenres.22853.r98672

**Published:** 2024-09-24

**Authors:** Bhagya Thimmappa

**Affiliations:** 1 USDA Agricultural Research Service, Chatsworth, NEW JERSEY, USA

In the work "The genome sequence of Ramsons hoverfly, *Portevinia maculata*," the authors reported nuclear genome assembly, annotation, and mitochondrial genome assembly for the Ramsons hoverfly fly. Ramsons hoverfly is localized to the UK and reported to damage host plant bulbs. Reported assembly and annotation resources are trivial in understanding these flies further. Overall, the manuscript is well written.

**Minor comments:**

1. Sequenced fly is male or female?
2. It would be great to mention briefly how gene modules are predicted. Was RNA-Seq data used to support predicted gene modules? This will help the reader decide how good the gene prediction is.
3. Also, does the annotation have alternative splicing information?
4. It appears the BUSCO value mentioned is for the genome assembly. Please clarify this in the table and provide the BUSCO value for structural annotation so we have some idea of how good the structural annotation is.

Are sufficient details of methods and materials provided to allow replication by others?

Partly

Is the rationale for creating the dataset(s) clearly described?

Yes

Are the datasets clearly presented in a useable and accessible format?

Yes

Are the protocols appropriate and is the work technically sound?

Yes

Reviewer Expertise:

Genomics, Bioinformatics

I confirm that I have read this submission and believe that I have an appropriate level of expertise to confirm that it is of an acceptable scientific standard.

# Reviewer report: Reviewer response for version 1

**DOI:** 10.21956/wellcomeopenres.22853.r98673

**Published:** 2024-09-18

**Authors:** Komal Kumar Bollepogu Raja

**Affiliations:** 1 Department of Pathology & Immunology, Baylor College of Medicine, Houston, TX, USA

The manuscript by Crowley et al presents a comprehensive report on the genome sequencing and annotation of the Ramsons hoverfly ( *Portevinia maculata*). Details of assembly process, quality metrics, and genome annotation, offer a valuable resource for understanding the genetic basis of this species. The study includes high-quality genomic data with a scaffold N50 of 310.9Mb and a detailed annotation of around 25,000 genes. The quality metrics such as a Quality Value (QV) of 65.2 and BUSCO completeness of 96.9%, show the reliability of the genomic data. Overall, the manuscript is good, and I have some minor suggestions that aim to improve the manuscript.

1. I suggest adding a few sentences on how the genome does sequence enhance our understanding of *P*. *maculata’s* biology or ecology
2. The introduction provides good background on *P. maculate *but can benefit if more details on the importance of this study are included. A clear research objective sentence that would help frame the study.

Overall, the manuscript presents a valuable contribution to the field of genomics with a high-quality genome assembly and annotation of *Portevinia maculata*. Addressing the points mentioned above would strengthen the manuscript and enhance its impact.

Are sufficient details of methods and materials provided to allow replication by others?

Yes

Is the rationale for creating the dataset(s) clearly described?

Partly

Are the datasets clearly presented in a useable and accessible format?

Yes

Are the protocols appropriate and is the work technically sound?

Yes

Reviewer Expertise:

Single cell omics, Genomics, Bioinformatics, Sequencing, Developmental Neurobiology

I confirm that I have read this submission and believe that I have an appropriate level of expertise to confirm that it is of an acceptable scientific standard, however I have significant reservations, as outlined above.

# Reviewer report: Reviewer response for version 1

**DOI:** 10.21956/wellcomeopenres.22853.r98669

**Published:** 2024-09-18

**Authors:** Nikoletta Andrea Nagy

**Affiliations:** 1 University of Debrecen, Debrecen, Hungary

The manuscript is well written, and the genome presented is of high quality and can contribute to future research on hoverflies.

**Minor comments:**

- I would recommend referring to Figure 1 in the description of the species in the Background section.
- Include the sex of the specimen used for DNA extraction.

Are sufficient details of methods and materials provided to allow replication by others?

Yes

Is the rationale for creating the dataset(s) clearly described?

Yes

Are the datasets clearly presented in a useable and accessible format?

Yes

Are the protocols appropriate and is the work technically sound?

Yes

Reviewer Expertise:

Insect genomics

I confirm that I have read this submission and believe that I have an appropriate level of expertise to confirm that it is of an acceptable scientific standard.

# Reviewer report: Reviewer response for version 1

**DOI:** 10.21956/wellcomeopenres.22853.r98671

**Published:** 2024-09-17

**Authors:** Stephan Koblmüller

**Affiliations:** 1 University of Graz, Graz, Austria

In this data note, Crowley *et al*. present the genome assembly of Ramson’s hoverfly, a hoverfly species occurring in Northern and Central Europe. The background section gives a concise, but nonetheless informative, introduction to the study species. The data are clear, and the methods are well described. I have no objections to any of the data presented or the results. As far as I can tell, the datasets are deposited in publicly accessible repositories.

Are sufficient details of methods and materials provided to allow replication by others?

Yes

Is the rationale for creating the dataset(s) clearly described?

Yes

Are the datasets clearly presented in a useable and accessible format?

Yes

Are the protocols appropriate and is the work technically sound?

Yes

Reviewer Expertise:

phylogenetics-/genomics, population genetics/genomics

I confirm that I have read this submission and believe that I have an appropriate level of expertise to confirm that it is of an acceptable scientific standard.

# Reviewer report: Reviewer response for version 1

**DOI:** 10.21956/wellcomeopenres.22853.r98674

**Published:** 2024-09-11

**Authors:** Juan Wulff

**Affiliations:** 1 North Carolina State University, Raleigh, USA

The manuscript is well written, concise but clear enough, following the style of a short communication or a brief report. The background provided is sufficient to understand the model under study, and the methodology used was a combination of deep sequencing tools to obtain a high-quality genome assembly of *Portevinia maculata*. I recommend the authors improving the gene annotation in future studies.

Are sufficient details of methods and materials provided to allow replication by others?

Yes

Is the rationale for creating the dataset(s) clearly described?

Yes

Are the datasets clearly presented in a useable and accessible format?

Yes

Are the protocols appropriate and is the work technically sound?

Yes

Reviewer Expertise:

Entomology; Molecular Biology; Genetics

I confirm that I have read this submission and believe that I have an appropriate level of expertise to confirm that it is of an acceptable scientific standard.

# Reviewer report: Reviewer response for version 1

**DOI:** 10.21956/wellcomeopenres.22853.r90067

**Published:** 2024-08-12

**Authors:** Weir Jamie

**Affiliations:** 1 Institute of Ecology and Evolution, University of Edinburgh, Edinburgh, UK

This note covers the genome sequence of the Ramsons hoverfly *Portevinia maculata. *It is a well-written note with clear and sufficient biological and methodological background provided. I have only one minor edit to suggest, see below.

**MINOR COMMENTS:**

**Genome Sequence Report, line 1/2**: Make it clear these numbers are collection site co-ordinates, include "E" or "N" and degree symbol.

Are sufficient details of methods and materials provided to allow replication by others?

Yes

Is the rationale for creating the dataset(s) clearly described?

Yes

Are the datasets clearly presented in a useable and accessible format?

Yes

Are the protocols appropriate and is the work technically sound?

Yes

Reviewer Expertise:

Evolutionary ecology, entomology. Particularly expertise in the Lepidoptera (butterflies and moths).

I confirm that I have read this submission and believe that I have an appropriate level of expertise to confirm that it is of an acceptable scientific standard.
